# CHARGE Syndrome Associated With Persistent Hyperplastic Primary Vitreous: A Case Report

**DOI:** 10.1155/crop/2159237

**Published:** 2026-06-07

**Authors:** Li Liu, Liyun Guo, Zailin Xiao, Liwei Zhang, Xuan Ma, Wen Tang, Mei Liu, Shuai Xiong

**Affiliations:** ^1^ Yunnan Eye Institute & Key Laboratory of Yunnan Province, Yunnan Eye Disease Clinical Medical Center, Affiliated Hospital of Yunnan University, Yunnan, China, ypfph.com; ^2^ Kunming Aier Eye Hospital, Kunming, Yunnan, China

**Keywords:** CHARGE syndrome, CHD7 mutation, coloboma, congenital cataract, persistent fetal vasculature

## Abstract

**Background:**

CHARGE syndrome is a rare genetic disorder caused primarily by CHD7 mutations, affecting multiple organs, including the eyes, heart, and ears. Ocular abnormalities are common, but bilateral persistent fetal vasculature (PFV) has not been previously reported in CHARGE syndrome. PFV results from the failure of the fetal hyaloid vasculature to regress, typically affecting one eye.

**Case Presentation:**

We report a 10‐month‐old female with bilateral PFV associated with CHARGE syndrome, confirmed by a de novo CHD7 mutation. Systemic evaluation revealed sensorineural hearing loss and congenital cardiac defects. The patient subsequently underwent cataract extraction and intraocular lens implantation in the left eye. Postoperatively, a partial improvement in esotropia was observed.

**Conclusions:**

This case represents the first documented instance of bilateral PFV in CHARGE syndrome. It expands the known ocular manifestations of the syndrome and underscores the importance of early genetic diagnosis and multidisciplinary care to optimize patient outcomes.

## 1. Background

CHARGE syndrome (CS) is a rare autosomal dominant genetic disorder [1, 2], with its acronym representing key features: Coloboma of the eye, Heart defects, Atresia of the choanae, Retardation of growth and development, Genital hypoplasia, and Ear anomalies, including hearing loss [3]. The syndrome affects approximately 1 in 12,000 live births, with mutations in the CHD7 gene on chromosome 8q12 identified as the primary cause in 67%–90% of cases [4–6]. CHD7 plays a crucial role in chromatin remodeling, regulating gene expression across various tissues during early development. The wide range of clinical manifestations in CS stems from this genetic dysfunction, resulting in significant variability in symptom severity and combinations among affected individuals.

As a multisystem disorder, CS affects several organs, including the eyes, heart, ears, craniofacial structures, and nervous system [7–9]. The complexity and variability of congenital anomalies often make early diagnosis challenging. Ocular manifestations, which are frequently bilateral and asymmetric, commonly include colobomas, microphthalmia, cataracts, strabismus, and nystagmus [6, 10–12]. However, persistent fetal vasculature (PFV), also known as persistent hyperplastic primary vitreous (PHPV), is rarely observed in CS, particularly in its bilateral form. PFV is a congenital condition resulting from the incomplete regression of the fetal hyaloid vasculature during ocular development. Although typically monocular, PFV has been associated with other congenital syndromes, such as trisomy 13, Aicardi syndrome, and Walker–Warburg syndrome. Bilateral PFV, especially in the context of CS, is exceedingly rare and represents a novel clinical finding.

We describe a case of bilateral PFV in a child with CS. This report reviews the clinical findings, including ocular anomalies and genetic testing results, and explores their connection to the pathogenesis of CS.

## 2. Case Presentation

A 10‐month‐old female infant was referred to the Affiliated Hospital of Yunnan University in May 2023 due to a one‐month history of leukocoria in the left eye and failure to fix and follow light or objects in both eyes. She was born at full term, weighing 3.32 kg, with no notable complications during delivery, although the Apgar score was not recorded.

A comprehensive systemic evaluation was performed prior to surgery. Neonatal hearing screening revealed sensorineural hearing loss in the right ear. Cardiac ultrasound demonstrated an atrial septal defect and patent ductus arteriosus, which were associated with intermittent cyanosis during crying. Developmental delay was noted, particularly in motor skills and feeding, with frequent choking episodes. Nasopharyngoscopy at 2 months of age revealed rhinitis and laryngeal tenderness. Cranial MRI showed bilateral widening of the frontotemporal extracerebral space.

The child’s parents reported no family history of genetic disorders, and both parents were healthy. There was no consanguinity, and the pregnancy had been uncomplicated. Despite being diagnosed with bilateral congenital cataracts and iris defects, surgical intervention was deferred due to the child’s feeding intolerance, low body weight, and elevated anesthetic risk.

At 7 months, the patient was hospitalized for a high fever and respiratory distress, initially suspected to be caused by a coronavirus infection. Further investigation revealed acute laryngitis, severe laryngeal obstruction, and associated myocardial and hepatic dysfunction. By 9 months of age, cataract surgery on the left eye was performed at Affiliated Hospital of Yunnan University.

### 2.1. Preoperative Examination

**Figure 1:**
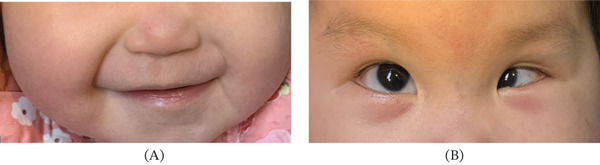
External appearance and ocular alignment. (A) Facial asymmetry with a shallow left nasolabial fold. (B) Facial asymmetry with a shallowed left nasolabial sulcus.

Upon admission, the infant appeared alert but displayed notable facial asymmetry, with a shallow nasolabial fold on the left side (Figure 1A). Low‐set, deformed auricles were observed bilaterally. Ocular examination was limited due to poor cooperation. The left eye was significantly smaller than the right, and corneal light reflex testing at 33 cm showed approximately 35° esotropia of the left eye (Figure 1B). The patient was unable to fixate or follow targets bilaterally, and horizontal nystagmus was present in both eyes. Intraocular pressure was within normal limits, but detailed posterior segment examination was not feasible.

### 2.2. Systemic Evaluation

Auditory testing demonstrated sensorineural hearing loss in the right ear, confirmed by otoacoustic emissions (TEOAE) and auditory brainstem response (ABR) testing. Cardiac ultrasound revealed an atrial septal defect (2.8 mm), patent ductus arteriosus (2.6 mm), and patent foramen ovale. Ultrasound examination of the urinary and reproductive systems showed no abnormalities. Cranial MRI demonstrated bilateral widening of the frontotemporal extracerebral space, suggestive of brain underdevelopment.

### 2.3. Imaging and Diagnostics

Under general anesthesia, operating microscope examination revealed bilateral iris defects and lens clouding in both eyes, with the right eye showing inferior lamellar lens opacity and the left eye showing central clouding. Axial lengths measured 22.03 mm in the right eye and 20.26 mm in the left eye. Central corneal thicknesses were 570 *μ*m in the right eye and 625 *μ*m in the left eye. Corneal diameters measured 10.5 mm horizontally and 10 mm vertically in the right eye, and 9.5 mm horizontally and 9 mm vertically in the left eye.

### 2.4. Surgical Intervention

The patient underwent cataract extraction, posterior capsulotomy, anterior vitrectomy, and intraocular lens implantation in the left eye. Fibrovascular tissue extending from the posterior lens was observed, PFV is considered a possibility.

### 2.5. Postoperative Examination

**Figure 2:**
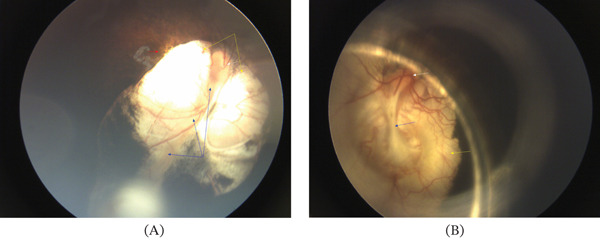
Postoperative RetCam III images. (A) Right eye with fibrous streaks extending into the vitreous cavity from the posterior lens capsule, connected to the optic disc (blue), with large choroidal defects around and beneath the optic disc (yellow). The morphology of the macula (red) and disc (white) is visible. (B) Left eye showing Optic disc (white), retinal folds, fibrous proliferation (blue) and large choroidal defects (yellow). The macula and disc are not clearly visible.

**Figure 3:**
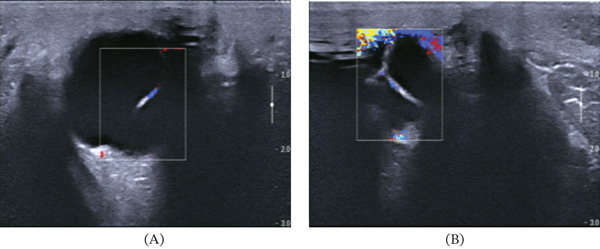
Color Doppler ultrasound images. (A) 2D ultrasound and color Doppler flow imaging (CDFI) of the right eye shows a thin, medium echogenic band connected from the posterior lens to the optic disc, in an “I” shape. (B) 2D ultrasound and CDFI of the left eye show a similar echogenic band. Short linear blood flow signals are visible within the band on CDFI.

At 1 month postoperatively, Postoperative RetCam III imaging confirmed the presence of a fibrovascular stalk extending from the posterior lens toward the optic disc was observed, consistent with PFV. Retinal folds and tractional changes were noted, optic disc hypoplasia with choroidal coloboma. Significant choroidal defects were identified, particularly around the optic disc in both eyes. Retinal folds were noted in the left eye, with the morphology of the macula and optic disc visible in the right eye (Figure 2A,B). Color Doppler ultrasonography revealed an echogenic band extending from the posterior lens capsule toward the optic disc. Short linear blood flow signals were detected within the band on Doppler imaging in both eyes. (Figure 3A,B). Optometric examination revealed +4.00 DS/−4.00 DC × 180° in the right eye and +1.50 DS/+0.50 DC × 100° in the left eye.

### 2.6. Genetic Testing

**Figure 4:**
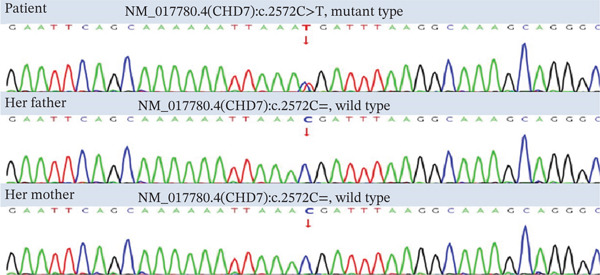
Sanger sequencing results showing a heterozygous CHD7 mutation in the proband, absent in both parents. Arrows indicate the mutation site.

Whole‐exome sequencing revealed a heterozygous nonsense mutation in the CHD7 gene (c.2572C>T, p.Arg858Ter) located in exon 8. This mutation results in premature termination of protein synthesis and was determined to be a de novo mutation, as it was absent in both parents (Figure 4).

## 3. Discussion

To our knowledge, this is the first reported and documented instance of bilateral PFV in a patient with CS, extending the known spectrum of ocular manifestations associated with this disorder. PFV, which typically presents unilaterally, results from the failure of the fetal hyaloid vascular system to regress, and its bilateral occurrence in this context is rare [13]. While bilateral PFV has been reported in other congenital syndromes [14–17], its presence in CS represents a novel clinical finding [18].

The presence of PFV in this patient, confirmed alongside a CHD7 mutation, suggests that PFV may be an underrecognized feature of CS. The involvement of neural crest cells in ocular development, particularly in structures such as the cornea, iris, and optic nerve [19, 20], further supports the connection between CHD7 mutations and ocular anomalies. Disruptions in neural crest cell migration and differentiation have been implicated in other features of CS, suggesting a shared developmental pathway for the ocular and systemic anomalies observed in this patient.

Early surgical intervention in this case, including cataract extraction and posterior capsulotomy, may reduce the risk of complications such as retinal detachment and secondary glaucoma, although long‐term follow‐up is required. Partial improvement in esotropia following corrective lenses suggests that early intervention may contribute to visual development. The lack of obstruction in the central optic axis and the absence of severe complications provide cautious optimism for improved visual outcomes in this patient.

The complexity of CS underscores the need for a multidisciplinary approach to care. In this case, continuous monitoring of cardiac, auditory, and developmental parameters will be crucial to managing long‐term complications. The patient’s atrial septal defect, patent ductus arteriosus, and sensorineural hearing loss emphasize the importance of early and ongoing intervention to prevent additional morbidity. Because of the patient’s poor preoperative cooperation and the cataract impairing visualization of the posterior segment, the possibility of PFV was not considered; therefore, combined posterior segment vitrectomy was not performed. Long‐term complications such as glaucoma, retinal detachment, and amblyopia remain important concerns and require close follow‐up.

Genetic testing for CHD7 mutations should be a routine part of the diagnostic workup in suspected CS cases. Early identification of the mutation allows for tailored management of the syndrome’s various manifestations and provides essential information for family counseling. This case illustrates the importance of comprehensive genetic and clinical evaluations in children with multisystem anomalies, where early diagnosis and intervention are keys to improving quality of life and reducing morbidity.

## 4. Conclusions

To our knowledge, this case report presents the first reported instance of bilateral PFV in a child with CS, expanding the known spectrum of ocular manifestations associated with this disorder. This finding highlights the importance of careful ocular evaluation in patients with CS and suggests that PFV may be an underrecognized feature. Early recognition and timely intervention, together with genetic testing and multidisciplinary management, may help optimize clinical outcomes.

**Nomenclature**
ABR: auditory brainstem response
CDFI: Color Doppler flow imaging
CS: CHARGE syndrome
CHD7: chromodomain helicase DNA‐binding protein 7
DC: Diopters cylinder
DS: Diopters sphere
IOL: intraocular lens
MRI: magnetic resonance imaging
PFV: persistent fetal vasculature
PHPV: persistent hyperplastic primary vitreous
TEOAE: transient evoked otoacoustic emissions

## Author Contributions

Li Liu and Liyun Guo contributed to literature search, case reporting, tables, writing, and revising the manuscript. Liwei Zhang and Xuan Ma contributed to literature search, case reporting, and revising the manuscript. All authors contributed to study design, data collection, editing and final approval of the manuscript. Li Liu and Liyun Guo contributed equally to this work.

## Funding

This study was supported by The Yunnan University Medical Research Fund Project, YDYXJJ2025‐0010; Yunnan Provincial Department of Education Science Research Fund Project, 10.13039/501100019413, 2025J0032; Yunnan Science and Technology Plan Project, 202605AF350044.

## Disclosure

All authors have read and approved the manuscript.

## Ethics Statement

Written informed consent to participate was obtained from the patient’s parents.

## Consent

Written informed consent for publication of clinical details and images was obtained from the parents of the patient.

## Conflicts of Interest

The authors declare no conflicts of interest.

## Data Availability

The data that support the findings of this study are not publicly available due to their containing information that could compromise the privacy of patient but are available from the corresponding author upon reasonable request.
